# Analyses and Declarations of Omega-3 Fatty Acids in Canned Seafood May Help to Quantify Their Dietary Intake

**DOI:** 10.3390/nu13092970

**Published:** 2021-08-26

**Authors:** Peter Singer, Volker Richter, Konrad Singer, Iris Löhlein

**Affiliations:** 1 European Omega-3 Council, 60598 Frankfurt am Main, Germany; info@ak-omega-3.de; 2 Institute of Laboratory Medicine, Clinical Chemistry and Molecular Diagnostics, University of Leipzig, 04103 Leipzig, Germany; vwrichter@gmx.de; 3 A-Connect Consulting, Sao Paulo 01311-200, SP, Brazil; konrad.singer@a-connect.com

**Keywords:** omega-3 fatty acids, canned seafood, coldwater fish, regional and seasonal variations of fatty acids, declarations of quantity, mixed concepts, CVD

## Abstract

The American Heart Association (AHA) recently confirmed common recommendations of one to two fish dishes per week in order to prevent cardiovascular disease (CVD). Nevertheless, the natural fluctuations of lipids and fatty acids (FA) in processed seafood caught little public attention. Moreover, consumers of unprocessed seafood in general do not know how much omega-3 fatty acids (omega-3 FA) within servings they actually ingest. The few studies published until today considering this aspect have been re-evaluated in today’s context. They included four observational studies with canned fatty coldwater fish (mackerel and herring from the same region, season, producer and research group). Their outcomes were similar to those conducted in the following years using supplements. Cans containing seafood (especially fatty coldwater fish) with declared content of omega-3 FA are ready-to-use products. Human studies have shown a higher bioavailability of omega-3 FA by joint uptake of fat. Canned fatty coldwater fish contain omega-3 FA plus plenty of fat in one and the same foodstuff. That suggests a new dietary paradigm with mixed concepts including several sources with declared content of omega-3 FA for reducing the cardiovascular risk and other acknowledged indications.

## 1. Introduction

### 1.1. Role and Feature of Seafood as Sources of Long-Chain Omega-3 FA

Previous studies investigating fish consumption as a source of omega-3 FA have inconsistently shown beneficial effects on cardiovascular risk factors, CVD incidence and mortality [1,2,3,4,5,6,7,8]. In a pooled analysis of four large cohort studies from 58 countries based on questionnaires [9], fish consumption of at least 175 g (corresponding to two servings weekly) reduced CVD in high-risk individuals (secondary prevention) but not in individuals with no signs of CVD (primary prevention). Oily fish was more effective [9]. The cardiovascular effectiveness of seafood was summarized in great detail by the Science Advisory from the AHA [10]. The authors focused their review on seafood which represents the primary source of long-chain omega-3 FA. They confirmed that one to two seafood meals per week are suitable to reduce the risk of CVD, ischemic stroke, and sudden cardiac death. Moreover, seafood concomitantly replaces the dietary intake of less healthy food rich in saturated and/or omega-6 FA. These guidelines for the dietary intake of omega-3 FA in seafood should be considered in more detail from the quantitative point of view.

Fish oil supplements are not the focus of this review. They undoubtedly remain the most common sources of omega-3 FA and cannot be ignored in context with the actual topic. A comprehensive review summarized recently their relevance for primary and secondary prevention of CVD [11]. Encapsulated fish oil concentrates have a standardized content of omega-3 FA of more than 30% as TG or ethyl esters (high concentrates as prescribed drugs containing up to 85%). Consequently, fish oil supplements permit defined dosages of omega-3 FA. On the other hand, fatty coldwater fish (in its edible portions) contain variable amounts of only 5–15% of omega-3 FA in their TG fraction.

Although experts and scientific boards for fish consumers equivocally recommend a habitual intake of one to two fish dishes per week, definable amounts of omega-3 FA within the servings are unknown. Uncertainties exist due to seasonal and regional variations of fat, TG and FA even within the same fish species [12,13]. In the North Atlantic Region at the end of a year, TG, eicosapentaenoic acid (EPA), and docosahexaenoic acid (DHA) are significantly higher compared with the beginning of a year (Table 1).

These variations are relevant and need to be taken into account [14]. They are able to explain comprehensible differences in amounts of TG and FA between the same fish species listed in various provisional tables [15,16]. So far, desired doses have to be estimated from tables prior to consumption. Therefore, an unreflecting adoption of such information seems questionable.

### 1.2. Preference of Coldwater Fish in Canned Products

Coldwater fish from the polar regions is especially rich in long-chain omega-3 FA. The most common species (herring, salmon, blue-fin tuna, mackerel, anchovies, sardines) are also available in canned products and as deep-frozen food. So far, however, a disadvantage was the lack of information regarding their actual content of omega-3 FA. Lobster, shrimps, scallops, oyster, cod, tilapia, freshwater fish, as well as their canned products, have lower levels of long-chain omega-3 FA. Similarly, seafood derived from coastal regions and aquaculture contains less EPA and DHA compared with coldwater fish. That also holds for their canned products. Knowing the actual FA composition of fish lipids is an important prerequisite for dietary studies. Omega-3 FA hold only the third place after monoenoic and saturated FA within the range of the groups of FA in coldwater fish (Table 2).

Despite this minor position, long-chain omega-3 FA provide their multifarious biochemical effectiveness. From this point of view, saturated and monounsaturated FA in coldwater fish are of minor biological importance for a healthy diet and its biochemical benefits [17,18].

The Regulations of the European Parliament and the Council of Nutrition and Health [19] include declaring the amounts of omega-3 FA in seafood. In consequence, cans declaring their contents of saturated and unsaturated FA—recently likewise with the additional level of omega-3 FA—are increasingly available on the European market. This allows finding the definite dose of these FA in canned products recommended for dietary intake by the European Food Safety Authority (EFSA) [20,21] and national scientific societies (see below). Their recommendations mostly suggest 250 mg/day for primary prevention and higher doses for hypertriglyceridemia, hypertension and secondary prevention of CVD.

### 1.3. Reason for a Reevaluation

Canned seafood was an irrelevant source of EPA and DHA for decades since the beginning of the omega-3 research, even before fish oil capsules were introduced. No systematic clinical studies have been found in the literature so far. In order to design such trials using seafood, it is desirable to know both the quantity of omega-3 FA within the body of the individual consumers as well as in the servings to be consumed. Therefore, studies based on canned coldwater fish were designed to investigate the widely unknown clinical effects of omega-3 FA at that time. That gave reason to conduct a reevaluation of those previous studies and their outcomes in today’s context, and more precisely define the recommendations of the AHA [10].

## 2. Similar Methods in Four Clinical Studies

For scientific purposes, it was necessary to analyze the content of FA within cans using laboratory tests prior to planned clinical studies. Therefore, mackerel and herring fillet in tomato pulp, respectively, were selected for examinations by gas-liquid chromatography and other tests. The analyses of mackerel and herring fillet, respectively, and tomato pulp revealed a high percentage of long-chain monoenoic FA (gadoleic acid—C20:1 and cetoleic acid—C22:1). They are typical for coldwater fish in the Arctic regions (Table 2). Their biological meaning remains unclear [17,18]. Together with other monounsaturated FA they are accumulated mainly in the TG fraction as compared with phospholipids (PL). Omega-3 FA in the PL fraction were obviously higher than those in TG. However, the quantity of PL was much lower than that of TG [18]. Consequently, TG were the most important lipids within canned fish for the planned studies. The percentages of omega-6 and omega-3 FA in tomato pulp added to the fish fillet within the cans were remarkably high as well. The probable reason was an exchange between fish fillet and pulp while they were stored together within the cans. Therefore, it should be recommended to consume the whole content of the cans and not only the fish fillet itself.

Prior to a series of four observational studies, 4000 cans of mackerel fillet in tomato pulp and 1200 cans of herring fillet in tomato pulp (200 g net weight each) were purchased from the same manufacturer (Fischkombinat Rostock, Rostock, Germany). They derived from one catch in the same region (North Atlantic) and season [18]. The contents of cans with mackerel fillet in tomato pulp (analyses in triplicate) amounted to 27.0 g TG, 1.0 g PL, 1.1 g EPA, 1.4 g DHA (together 2.5 g long-chain omega-3 FA per can). The contents in cans with 200 g herring fillet in tomato pulp amounted to 25.0 g TG, 1.2 g PL, 0.5 g EPA, 0.8 g DHA (together only 1.3 g long-chain omega-3 FA). The amounts of EPA plus DHA in the canned herring described were lower than those, e.g., found in canned herring by another research group: 1.8 g/100 g = 3.6 g/200 g per can [22].

All studies were carried out in a research clinic (Dept. of Clinical Lipid Research, Central Institute of Cardiovascular Research of the Academy of Sciences, Berlin, Germany). The participants consumed the whole content of two cans of mackerel fillet in tomato pulp or herring fillet in tomato pulp per day (one in the late morning, the other in the evening) over two weeks within an isocaloric diet. Hence, the daily amounts were for mackerel 2 × 2.5 g/can = 5.0 g/day of EPA plus DHA and for herring 2 × 1.3 g/can = 2.6 g/day EPA plus DHA (i.e., half as much). The participants tolerated the unusually high intake of canned fish without complaints. No bleeding tendency was observed [18].

The daily amounts of omega-3 FA ingested during the short period of two weeks were similar to those (5 g/day) accepted as being safe by the EFSA [21]. Tests of longer durations with those amounts were considered unreasonable for the participants. Dietary interviews, blood pressure measurements and blood samples (including lipids, lipoproteins and FA profiles) were taken before and at the end of the dietary periods as well as three months later after the return to habitual diet (control). The subjects were requested to keep their normal physical activity and to reduce their daily fat intake by about 50 g to keep the amount of total fat on the same level. All procedures were supervised by two dieticians.

## 3. Effects on Cardiovascular Risk Factors

### 3.1. Results of Canned Mackerel and Herring in Healthy Volunteers

In a first preliminary study, healthy male volunteers were carefully randomized and put on a mackerel or herring diet (two cans/day) consisting of a prescribed isocaloric regimen in a cross-over design for two weeks [18]. At the end of the mackerel period with an intake of 5.0 g/day EPA plus DHA, serum TG, total cholesterol, plasma noradrenaline, systolic and diastolic blood pressure were significantly decreased (*p* < 0.01), whereas postheparinlipolytic activity (PHLA) was increased. The data returned to their initial levels three months after returning to habitual diet (control). The herring diet with a lower intake of 2.6 g/day of omega-3 FA showed similar but insignificant changes of all those parameters, indicating a dose-dependent effectiveness. These data of healthy volunteers were the reason for investigating patients with risk factors of CVD as well.

### 3.2. Results in Patients with Hyperlipoproteinemia (HLP)

**Figure 1:**
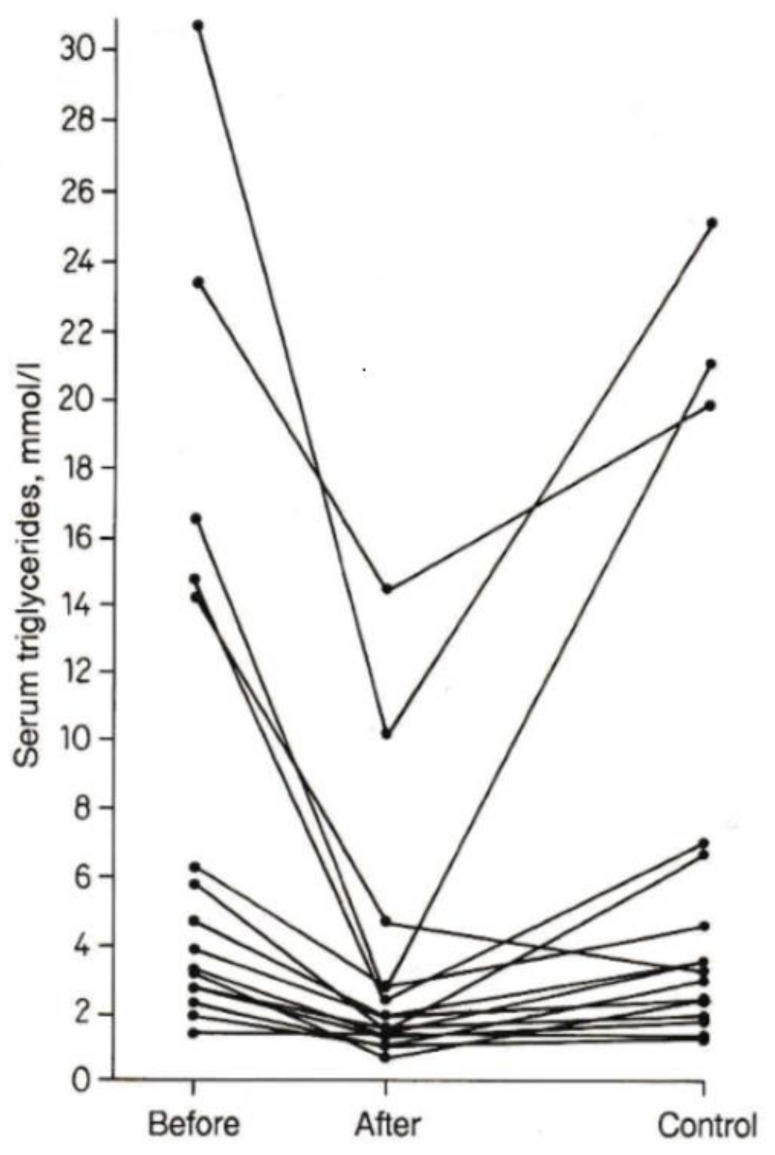
Serum TG of individual patients with HLP types IIa, IV and V before and two weeks after an isocaloric diet including canned mackerel (2 cans/day over 2 weeks), control 3 months later on habitual diet; modified after [25].

In a second pilot study, male inpatients with HLP types IIa, IV, and V—a common classification at that time [23]—were observed using the same study design [24]. An expanded version of the study confirmed that at the end of the mackerel diet, blood pressure, serum TG, total and LDL cholesterol were significantly reduced, returning nearly to the basal levels after subsequent three months on habitual diet [25]. When consuming the same doses of canned mackerel, decreases in the highest initial values of TG were more pronounced (Figure 1) according to Wilder’s law of initial value [26].

The standard deviations were extraordinarily large due to the marked variability of the initial values between individual patients [26]. The herring diet with the lower intake of omega-3 FA resulted in minor changes. In this case, for dieticians and health care professionals the follow-up of individual patients might be more illustrative instead of summarizing data from groups or cohorts [27]. This impressively can be seen in Figure 1. A prerequisite for this procedure is careful randomizations, especially if small groups are studied [18,25].

In large cohorts and meta-analyses, the summarizing data can mask the individual features of consumers and patients (see below). The TG levels returning to the initial values after finishing the mackerel period (control 3 months later) suggests the necessity of a long-term diet for controlling risk factors of CVD. In this way, therapists and consumers can avoid the rerise in TG and keep their decreased levels permanently low (compare Figure 1).

### 3.3. Canned Mackerel and Herring in Patients with Mild Essential Hypertension

The blood pressure-lowering effect in healthy volunteers and in patients with HLP gave the impetus for a further trial including male inpatients with mild essential hypertension using the same study design [28]. Systolic blood pressure was significantly lower at the end of the mackerel diet, returning to the basal levels three months later (control). At the end of the herring period with a lower intake of omega-3 FA blood pressure remained unchanged. Serum TG, total and LDL cholesterol were significantly decreased at the end of the mackerel and herring diets, whereas HDL cholesterol was increased only at the end of the mackerel diet.

During a standardized psychophysiological stress test (sentence completion tasks) systolic and diastolic blood pressure were significantly lower at the end of the mackerel period. The stress-dependent rise in thromboxane A_2_—at the beginning of the study—measured as its stable metabolite thromboxane B_2_ failed at the end of the period with canned mackerel. The changes in the herring period were of minor degree and not significant.

### 3.4. Long-Term Study: Canned Mackerel in Patients with Essential Hypertension

Two cans of mackerel or herring in tomato pulp per day over two weeks (corresponding to 5.0 g/day or 2.6 g/day EPA plus DHA, respectively), used in the preceding three short-term studies, were unrealistic for a long-term intake. Therefore, in a subsequent study [29] male outpatients with mild essential hypertension were put on a diet supplemented with three cans per week containing mackerel fillet in tomato pulp (corresponding to 7.5 g/week equivalent to 1.1 g/day EPA plus DHA) over 8 months. This dose in the long-term period was similar to that of about 1 g/day, which at present is recommended by scientific boards for secondary prevention (see below). At the end of the long-term period systolic and diastolic blood pressure were significantly decreased and returned to the initial values after a subsequent control period of two months. In a control group with randomized hypertensive patients on habitual diet no changes in blood pressure were seen.

## 4. Arguments for the Design of the Four Studies

The reevaluation of the above-mentioned studies is encouraged by the 2018 published Advisory from the American Heart Association (AHA) focused on seafood [10]. The reference to the previous results appears to be nostalgic. On the other hand, it was impossible to find similarly systematic trials from other research groups in the literature concerning clinical effects of canned seafood on cardiovascular risk factors. Thus, the results of the above-mentioned studies remain fully valid and are contemporary contributions of dietary strategies including seafood rich in omega-3 FA. The intention to design the studies based on canned coldwater fish (mackerel vs. herring) was to ascertain clinical effects of omega-3 FA apart from fresh fish with their quantitative uncertainties (see above). Fish oil concentrates were just at the beginning of their introduction as supplements. At that time—in the pioneering period of omega-3 research—in preliminary clinical trials the data from only small groups of participants were published [1,2,3,4,5,6,7,8].

It had been an advantage that all cans used came from the same region, season and manufacturer. The results of the mentioned studies indicated a benefit of canned coldwater fish similar to that of fish oil supplements thereafter commonly used as sources of omega-3 FA.

The large amounts of coldwater fish in the first three short-term studies have been chosen since the effects of omega-3 FA were widely unknown at that time. With increasing experience, the doses were lowered in the subsequent study.

Because of the extreme design the observational studies were carried out in groups of only a few participants until the level of significance (*p* < 0.01) was reached [18,25,28]. The authors were aware that the large amounts of canned fish (two cans/day over two weeks) and omega-3 FA (about 5 g/day) in the observational studies were unsuitable for a long-term diet and its recommendation. Nevertheless, the high doses were well tolerated with reliable compliance and no side effects or dropouts. They did not exceed the upper limit (safety up to 5 g/day) recently recommended by the EFSA [21]. From the above-mentioned data it can be confirmed that a daily intake of 5 g/day of omega-3 FA is safe at least for a short-term consumption. In conclusion, the limits of consuming omega-3 FA—minimal requirement: ~100 mg/day [30] and upper limit of 5 g/day [21]—provide a wide range of omega-3 FA for dietary practice.

## 5. Practical Aspects for Dietary Intake

**Figure 2:**
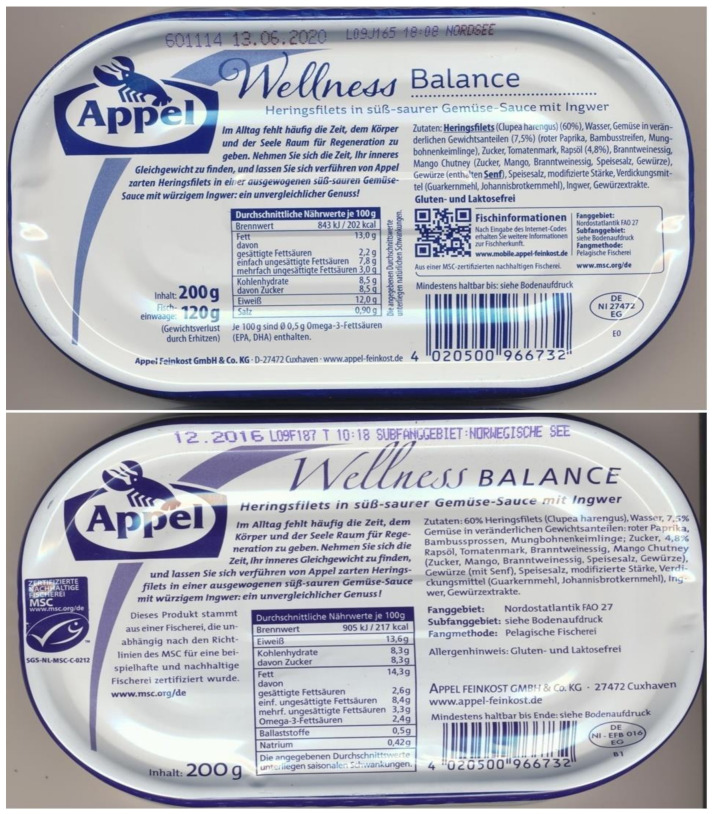
Example of two cans from the same manufacturer containing herring fillet in vegetable sauce derived from the same region (Northern Atlantic). Different amounts of omega-3 FA declared on the reverse side of the cans: 0.5 g/100 g (**above**) and 2.4 g/100 g (**below**)—presumably harvested and produced in different seasons (dates of fishing were uncertain), compare Table 1; random purchases in a German supermarket—from the years 2017 and 2019, respectively.

The additional declaration of omega-3 FA, apart from those indicating fat and saturated FA, on the labels of canned seafood would be helpful quantitative information for consumers and dieticians [21]. On the labels of now available cans, e.g., containing coldwater fish, omega-3 FA are usually declared within a range of 0.5–3.0 g/100 g. This reflects seasonal fluctuations even if they are harvested in the same region. An example provides the five-fold difference in the content of omega-3 FA declared on commercially available cans containing herring (Figure 2, compare Table 1).

The previous studies were carried out mainly with canned mackerel—at that time nicknamed ‘mackerel diet’. Nowadays, other coldwater fish such as herring, salmon and tuna are dominant in canned products on the European market.

Until recently, for consumers the intake of seafood was recommended by scientific boards without knowing their actual content of omega-3 FA [31]. It provided an uncertain source of TG and omega-3 FA. Comparison of Table 1 and Figure 2 illustrate a five-fold difference in coldwater fish probably depending on the season alone. This relevant divergence has to be considered.

Most recommendations until now define a dose between 250 mg/day and 500 mg/day for primary prevention and about 1 g/day for secondary prevention of CVD [31,32,33,34,35,36,37]. All of them are strikingly consistent within close limits [37]. None of them did consider the possibilities of individual requirements for consumers (see below). EFSA has recently proposed higher doses with different levels for various indications [21]. They can be tolerated without a risk of unwanted effects. About 2–4 g/day omega-3 fatty acids may contribute to the maintenance of normal blood TG levels and 3 g/day for maintenance of normal blood pressure. If necessary, changes in doses are possible at controls during follow-up. The daily intake of up to 5 g/day EPA plus DHA is considered safe at least for a short-term intake. It corresponds to the observational studies using canned mackerel as described above [18,25,28]. A trend towards higher doses is apparent. Recent recommendations suggest the possibility of higher doses of long-chain omeda-3 FA than those used in dietary practice and research so far [21,38,39,40,41]. This can be claimed for secondary prevention and treatment of CVD. Obviously, it is mainly indicated for dietary prevention and treatment of TG levels in hypertriglyceridemia [25,37,38], metabolic syndrome [37,39] and CVD as well [10,40,41]. A long-term consumption of omega-3 FA can include both seafood and fish oil supplements together at the same time. Both of them are acceptable as complements instead of alternatives. Following the arguments for declaring the content of omega-3 FA, the supply of canned seafood becomes more important than before considering dietary habits of fish consumers. In practice, the benefits and possible disadvantages must be balanced. The benefits surpass the disadvantages by far (Table 3).

According to existing recommendations, one to two cans/week of seafood is an acceptable amount. For instance, an intake of 2.5 to 5.0 g omega-3 FA with canned coldwater fish once per week is in line with the recommendations for primary prevention [10,21,31,32,33,34,35,36]. An intake of about 5.0 g/week of omega-3 FA equivalent to 0.7 g/day can easily be realized by replacing one or two recommended fish dishes per week [10] with one or two cans/week of coldwater fish. Considering the declared content within the cans gives a reliable dosage (compare Figure 2), in certain individual cases, more than the recommended one to two fish meals per week have been suggested [42]. In order to meet higher doses of omega-3 FA as advised by the EFSA [21], a concomitant intake of other marine products (deep-frozen fish, supplements of fish oil, algae oil, functional food) within a mixed concept is suggested [14,42]. It is advantageous if other sources (deep-frozen fish, supplements) contain declared quantities of omega-3 FA as well. If they complement the supply of omega-3 FA, or are preferred by consumers, the amounts of canned seafood can be flexibly adjusted. Some species of large fish (shark, swordfish, a.o.) and those from coastal regions are likewise sources of omega-3 FA, although their content is comparably low. They are less often available in canned products. These and other aspects support the suggestion of further analyses and consecutive declarations of various species of canned seafood.

## 6. Suggestions for Future Research

### 6.1. Comparison of Seafood with Fish Oil Supplements

It seems attractive to design studies comparing canned seafood and fish oil supplements with identical doses of omega-3 FA. Such comparisons are important from the quantitative point of view. In female volunteers, consumption of equal doses of EPA plus DHA (485 mg/day) from fatty fish (two servings of fresh, but no canned salmon or tuna per week) versus fish oil supplements (1–2 capsules/day) over 16 weeks resulted in similar changes without differences between FA and their effects in blood [43].

These data were different from other trials. In a previous preliminary study [44] in patients with HLP the intraindividual comparison of canned mackerel versus encapsulated fish oil adapted to a higher dose of EPA (2.2 g/day) has shown decreases in serum TG, free fatty acids (FFA), LDL cholesterol, apolipoprotein B and blood pressure in both groups of participants. The changes were, however, more pronounced at the end of the mackerel diet as compared with the supplements. This was associated with a higher bioavailability of EPA and DHA in the period of consuming the mackerel diet. Perhaps the differences between the findings in both studies resulted from higher daily doses of omega-3 FA and/or their uptake in the form of fatty meals with the canned mackerel in the latter [44].

**Figure 3:**
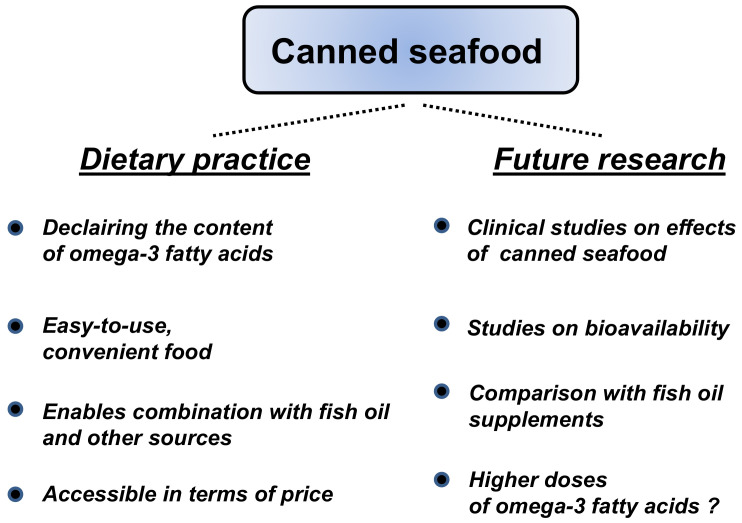
Suggestions of canned seafood being suited to dietary practice and future research.

In another study, consumption of fresh fish was superior at positively changing lipid profiles compared with fish oil, which suggested translations in the occurrence of cardiovascular events [45]. In human studies [46,47,48,49] fish meals (not canned) once or twice per week revealed higher levels of EPA and DHA in plasma and blood cells compared with daily fish oil supplementation. Possibly, the inclusion of omega-3 FA in fatty meals has modified their uptake and bioavailability. This can be expected with canned seafood as well. Presumably, several nutrients or constituents (probable lipids) of seafood other than omega-3 FA (compare Table 3) and the matrix of various foodstuff influences the bioavailability [50]. These data may contribute to a platform for future human studies (Figure 3).

Consumers can be encouraged to habitually ingest the content of one or two cans of fatty coldwater fish as a spike intake of omega-3 FA instead of weekly meals with other low-fat seafood.

In another context, Italian commercial canned blue-fin tuna in 50 g portions was equivalent to one capsule of omega-3 ethylester (1 g) is potentially more effective for a significant cell protection against oxidative stress [51]. Thus, canned blue-fin tuna was recommended as a functional food for the prevention of CVD.

### 6.2. Different Efficacy on High and Low Initial Levels of TG and Blood Pressure

High initial values of serum TG have shown a higher reduction compared with lower initial values using the same doses of omega-3 FA (Figure 1). Patients with HLP on a high-dose mackerel diet (2 cans/day equivalent to 5 g/day of omega-3 FA over two weeks) were subdivided according to their level of systolic blood pressure [52]. Higher initial values of systolic blood pressure (>160 mm Hg) saw a more pronounced decrease (minus 17 mm Hg) as compared with lower initial values (<160 mm Hg), resulting in a decrease of only 7 mm Hg. These data indicated that the intake of equal doses of omega-3 FA could more effectively decrease both high levels of basal blood pressure and TG compared with normal or low levels [25,26].

### 6.3. Justification for Quantifying the Supply of Omega-3 FA

A worldwide deficiency of essential omega-3 FA in populations and individuals can be presupposed. This is especially obvious in regions preferring a Western diet [53]. It can be minimized only by a long-term consumption of adequate and acceptable doses of these FA. The deficit can be controlled by quantifying procedures:

- Measurement of the status of fatty acids by means of the Omega-3 Index before and during follow-up.
- Adequate dosage of fish oil or algae oil supplements.
- Consumption of seafood and/or functional food with declared contents of omega-3 FA.

A combination of various dietary sources may be indicated according to personal preferences and circumstances.

### 6.4. Omega-3 Index and Controlled Intake of Omega-3 FA

Quantifying and declaring require control. The individual omega-3 FA status and its dietary optimizing can be exactly controlled by the Omega-3 Index with an optimum of 8–11% [54,55,56]. It enables an individual follow-up during long-term diet by measuring and optimizing the status of omega-3 FA. This is comparable with the common estimations of, e.g., blood sugar in diabetics, lipids in patients with HLP, and blood pressure in hypertensives [56]. Moreover, the knowledge of the omega-3 status is an important prerequisite for the interpretation of scientific studies. Low levels may have better prospects of a benefit. Ignoring this aspect may have been one of the reasons for neutral outcomes of dietary omega-3 FA in several recent intervention trials and meta-analyses [27,56,57].

Between different individuals the uptake, levels and bioavailability of omega-3 FA are variable [50,55]. Uptake and bioavailability are favored if omega-3 FA are jointly consumed with fat-containing meals [56]. In practice, it can be realized if EPA and DHA are consumed within, e.g., fatty fish (including canned fatty coldwater fish) instead of lean seafood. From this point of view, canned fatty coldwater fish containing EPA plus DHA and plenty of eatable fat (TG and phospholipids of the fillet) within one and the same foodstuff are candidates for increasing the bioavailability of omega-3 FA.

An Omega-3 Index was not considered in the above-mentioned AHA recommendations [10]. Different from that review, in a recent study based on fish-related dietary questionnaires [42], the consumption of at least three fish meals per week plus supplements of EPA + DHA markedly increased the Omega-3 Index to the target level of >8%. This is in line with the suggestion of mixed concepts already mentioned above. The effects of two meals per week (similar to those of most recommendations) or taking supplements were lower [42]. These data correspond with the recent tendency toward higher doses of omega-3 FA for several indications [21,37,38,39,40,41]. The best way of optimizing the omega-3 status remains testing the Omega-3 Index [42,56]. For future research, more controlled studies and recommendations considering the FA status by means of Omega-3 Indices are warranted. Including canned seafood by measurement and declaration of their omega-3 FA content may help to quantify the outcomes. In a current meta-analysis [57], a dose–response showed that an increase in fish consumption of 20 g/day—without considering fish species, processed products and their content of omega-3 FA—resulted in a 4% reduction in incidence and mortality of CvD, respectively. More systematic trials are needed to quantify the consumption of omega-3 FA and its effects on a scientific standard (Figure 3).

## 6.5. Acknowledgement by Consumers

In general, improvements in foodstuff labelling can provide a significant contribution to find specific food within a healthy overall diet. Regulations and labelling may intensify a general discussion on the relevance of quantifying certain nutrients, including canned fish, for dietary practice and research [58,59,60,61,62]. The described data support the significance of declaring the contents of omega-3 FA in canned marine products and their importance for the large field of their multifarious indications. Manufacturers of canned seafood should be encouraged to introduce or intensify declarations of omega-3 FA on their products. Canned seafood might be a recommendable component of a Mediterranean diet or other healthy eating patterns [63].

## 7. Conclusions

Apart from fish oil supplements, the recent AHA recommendations are the standard guideline for the dietary concept of seafood and omega-3 FA consumption. Fish consumers should know the actual contents of omega-3 FA in fresh and processed seafood, which are variable due to regional and seasonal fluctuations. Analyses and declaring the content of long-chain omega-3 FA in cans containing seafood—preferably fatty coldwater fish—help to quantify their supply. They are suitable to support and intensify future intentions for dietary intake under a new paradigm. The declarations of quantities should include omega-3 FA on labels of canned marine products in addition to fat, saturated and unsaturated FA, which in most cases are already declared. According to culinary habits, canned seafood might be consumed alone or jointly with other sources (e.g., deep-frozen seafood, encapsulated fish oil, algae oil, functional food), likewise with declared contents of omega-3 FA. Such information on canned seafood is valuable for mixed concepts within a healthy diet. In future research and quantified consumption, it may be important to know the actual content of omega-3 FA within various dietary sources as components of mixed concepts.

Recent clinical studies indicated a higher bioavailability of omega-3 FA if spike meals of seafood per week were compared with daily fish oil supplements. Moreover, apart from declaring the content of omega-3 FA, canned coldwater fish is rich in other lipids (TG and phospholipids), which per se favor the bioavailability of EPA and DHA. A quantification is possible by means of the Omega-3 Index. A low level might increase the chance of an effective diet or supplementation. An additional advantage of canned products is their rich availability on the market. Canned seafood meets the tendency toward convenience food. They are easy to transport and store, inexpensive and accessible for large parts of the population. The defined content of omega-3 FA in canned seafood makes it attractive for future dietary studies on their multifarious beneficial effects. It could be interesting to combine previous and actual data in common concepts for research and practice.

## Figures and Tables

**Table 1:** Content of TG, EPA and DHA in TG (g/100 g) of fresh coldwater fish from the North Atlantic (region near Newfoundland) during different seasons.

Fish Species	Month	TG	EPA	DHA	EPA + DHA
Herring	April	1.6	0.04	0.04	0.08
	September	7.3	0.67	0.72	1.39
Mackerel	February	2.4	0.11	0.12	0.23
	November	11.0	1.10	1.58	2.68

Analyses (means of triplicate) of fresh fish fillet by gas liquid chromatography—regardless of the studies to be discussed below; modified after [14].

**Table 2:** Saturated, monounsaturated and polyunsaturated FA (%) of TG and phospholipids (PL) of canned mackerel fillet pickled in tomato pulp, modified after [17].

	Mackerel		Tomato Pulp	
	TG	PL	TG	PL
**Monounsaturated FA**				
**16:1**	4.6	1.5	3.4	0.7
**18:1**	22.2	14.8	20.2	18.3
**20:1**	9.5	5.5	9.6	8.8
**22:1**	16.3	5.7	24.7	17.2
**Polyunsaturated FA**				
**Omega-6 FA**				
**18:2**	2.9	4.9	7.5	8.5
**20:4**	0.5	1.2	0.4	0.5
**Omega-3 FA**				
**18:3—(ALA)**	2.1	0.8	1.8	1.8
**20:5—(EPA)**	4.4	6.0	3.4	3.7
**22:5—(DPA)**	0.2	0.5	0.0	0.1
**22:6—(DHA)**	7.0	15.7	4.8	6.6
**Total of FA groups**				
**Saturated FA**	25.2	26.0	18.3	14.9
**Monounsaturated FA**	54.0	52.6	59.1	58.4
**Omega-6 FA**	3.4	6.1	7.9	9.0
**Omega-3 FA**	13.7	23.0	10.0	12.2

Analyses in triplicate, mean values; selected FA—therefore, the total values of the FA groups can be different from the sum of the individual FA. PL = phospholipids, ALA = alpha-linolenic acid, DPA = docosapentaenoic acid.

**Table 3:** Benefits or disadvantages of canned cold-water fish as sources of omega-3 FA.

Fish Species
**Benefits**
- Healthy natural food. - Easy-to-use products. - Abundantly available in supermarkets. - Meets the trend toward convenience food. - Available with common species of coldwater fish (herring, salmon, mackerel, tuna). - Easy to transport for fast meals (e.g., snacks, breaks, picnic a.o.). - Favors an increased uptake and bioavailability of EPA and DHA within fatty meals. - Declaration of the content of omega-3 FA (in g/100 g) on packaging and cans. - Enables quantifying the dietary intake of omega-3 FA in practice and science. - Ability to replace an excess of food with much saturated FA (e.g., meat, sausage, cheese). - Ability to replace an excess of omega-6 FA (food rich in linoleic acid). - Easy to store without refrigeration. - Requires no food preparation. - No necessity for major changes in eating habits. - Accessible in terms of price.
**Disadvantages**
- Not suitable for sophisticated meals. - Possible aversion or allergy to fish consumption.

## Data Availability

Not applicable.
